# Analytical SA-HCISCF Nuclear Gradients from Spin-Adapted Heat-Bath Configuration Interaction

**DOI:** 10.1021/acs.jctc.5c00021

**Published:** 2025-04-07

**Authors:** Mihkel Ugandi, Michael Roemelt

**Affiliations:** Institut für Chemie, Humboldt-Universität zu Berlin, Brook-Taylor-Str. 2, Berlin D-12489, Germany

## Abstract

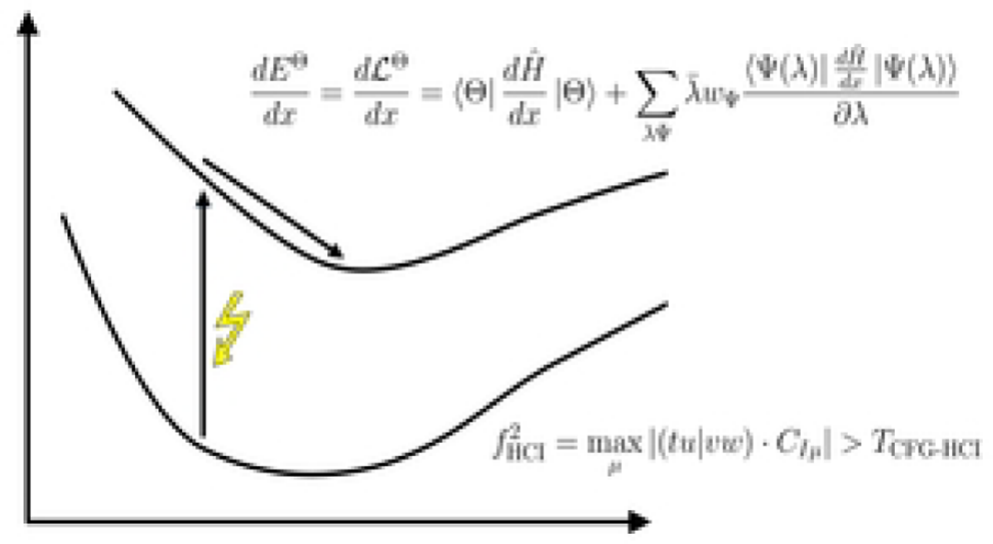

This work reports an implementation of the analytical nuclear gradients and nonadiabatic couplings with state-averaged SCF wave functions from a spin-pure selected configuration interaction (SCI) method. At the core of the implementation lies the evaluation of the Lagrange multipliers required for the variational calculation of the nuclear gradient. Using the same code infrastructure, we developed a fully CI-coupled second-order orbital optimization method. Both the calculation of the nuclear gradient and the second-order orbital optimization make use of density fitting in order to accelerate the calculation of the two-electron integrals. We demonstrate the use of analytical nuclear gradients in excited-state geometry optimizations for conjugated molecules. In addition, the first triplet excited-state geometry of a transition-metal catalyst, Fe(PDI), was optimized with up to 30 orbitals in the active space. Our results outline the capabilities of the implemented methods as well as directions for future work.

## Introduction

Multireference (MR) electronic structure methods constitute a valuable tool for studying chemical systems that exhibit static electron correlation effects. Examples of such effects can be found in computational studies of, e.g., transition metal chemistry,^1^ photochemistry,^2^ and catalysis.^3^ Traditionally, MR methods have been associated with large computational costs that scale exponentially with the size of the active orbital space. Yet, significant efforts and algorithmic developments over the last two decades have resulted in approaches that enable approximate full-CI (FCI) calculations on molecules with large active spaces. Well-known and established examples are the density matrix renormalization group (DMRG),^4−6^ FCI quantum Monte Carlo (FCIQMC),^7−9^ and selected-CI (SCI) methods.^10−15^ These CI solvers can be used together with orbital optimization to carry out approximate CASSCF calculations on molecules with very large active spaces.^16−20^ Furthermore, several recent studies combine large-CAS wave functions with multiconfigurational perturbation theory approaches to address dynamic electron correlation effects.^21−25^ Such methods hold the potential to yield quantitatively accurate results for chemical systems with many strongly correlated electrons.

Another important frontier for MR methods is the performance of geometry optimizations. Although density functional theory (DFT) approaches have long been established as the most popular tool for that purpose, multiconfigurational methods can offer reliable reference calculations in cases where DFT may struggle. Relevant examples include studying potential energy surfaces in transition metal catalysis and photochemical processes.^26^ In the latter case, it is often preferable to use the state-averaged CASSCF formalism, in which the orbitals are optimized for the electronic energy averaged across multiple states. For one, the state-averaging procedure provides a balanced description of multiple states, unlike the alternative state-specific approach. More importantly, state-averaged orbital optimization avoids the root-flipping problem, which may occur in the course of an excited-state geometry optimization.^27^ However, during the calculation of nuclear gradients, a difficulty arises with state-averaged wave functions. The energy of a specific state is not variational with respect to the orbital rotations. Taking an energy derivative directly would then lead to the appearance of nuclear response terms, which are prohibitively costly to evaluate. In the early 2000s, Stålring et al. developed an efficient approach for tackling this issue.^28^ Specifically, a variational SA-CASSCF energy Lagrangian was formulated that allows for the evaluation of the nuclear gradient without the need for any nuclear response terms. The only overhead is solving a set of equations for the Lagrange multipliers, which requires calculating the full electronic SA-CASSCF Hessian matrix elements. A decade later, this effort was followed by the works of Delcey et al. where density-fitting (DF) was introduced to accelerate the calculation of the two-electron derivative integrals.^29,30^ Snyder et al. formulated the equations for analytical SA-CASSCF nuclear gradients and nonadiabatic couplings in an atomic orbital basis. Their approach makes use of efficient screening of the two-electron integrals, which, combined with modern graphical processing units, enables calculations on systems of enormous size.^31,32^ The aforementioned advancements have brought large systems of chemical interest into the realm of feasibility for geometry optimization with MR methods. On the other hand, it is sometimes desirable to use active spaces that exceed what is feasible with the conventional CAS approach. Aiming in this direction, Freitag et al. reported the implementation of analytical SA-CASSCF nuclear gradients with DMRG wave functions.^33^ However, their implementation neglects the response of the rotation matrices in the matrix-product state. As a consequence, the errors in the gradient end up being comparatively large, even when large bond dimensions are employed. This limitation was later overcome by Iino et al., who developed an algorithm that employs a full Lagrangian for the state-averaged DMRGSCF ansatz.^34^ Very recently, Kim et al. put forth an implementation of the nuclear gradients with SA-SCISCF wave functions.^35^ Their approach makes use of the determinant-based adaptive sampling CI algorithm (ASCI).^13^ Remarkably, the implementation of Kim et al. uses the EN-PT2 perturbative energy correction in the excited-state nuclear gradient calculation.

In a recent work, we developed a heat-bath CI algorithm that works in the orbital configurational basis (CFG-HCI).^36^ The CFG-HCI (or HCI) algorithm is fully spin-adapted^37^ and capable of tackling considerably larger active spaces than FCI. This work extends the CFG-HCI method to the calculation of approximate SA-CASSCF analytical nuclear gradients and nonadiabatic coupling coefficients. A natural byproduct of this effort was the development of a fully CI-coupled second-order orbital optimization method, which was extensively used in this work. The performance of our implementation was significantly enhanced by employing density fitting in the calculation of two-electron integrals and their derivatives. In the next section, we will first give an overview of the theoretical concepts underlying the implementation: the HCI algorithm, the calculation of SA-HCISCF nuclear gradients with the DF approximation, and second-order orbital optimization. Then, the implementation is tested in a set of geometry optimizations. First, we will show how the approximate HCI nuclear gradients compare to the exact results from FCI. Then, we present results that demonstrate the accuracy-to-cost ratio in calculations involving up to 20 active orbitals. Finally, our results for the Fe(PDI) complex demonstrate the feasibility of using the reported method in geometry optimizations with active spaces beyond the exact CAS limit.

## Theory and Implementation

In the following, indices *i*, *j*, *k*, and *l* are used for internal orbitals; *a*, b, *c*, and *d* for external orbitals; *t*, *u*, *v*, and *w* for active orbitals; and *p*, *q*, *r*, *s*, *m*, *n*, and *o* for general molecular orbitals. Capital indices such as *I* and *J* are used to denote general many-body basis functions such as SDs or CSFs. When used together with Greek letters, e.g., μ and ν, the capital index denotes the orbital configuration part, and the Greek letter denotes the spin-coupling part of the CSF: |*I*μ⟩, |*J*ν⟩, etc. In the context of density fitting, the Greek letters μ, ν, κ, and τ denote the atomic basis functions, and the capital letters *P*, *Q*, *R*, and S denote the auxiliary basis functions. Throughout this work, we assume an exponential parametrization of both the orbital and configurational variable spaces of the SA-CASSCF wave function (and its approximate variants).^38^ More precisely, any state
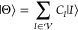
1can be transformed into state  via
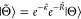
2

The orbital and state rotation operators are defined as
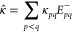
3and
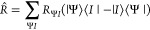
4where the summation over Ψ runs over the set of electronic states that is being averaged over, while *I* refers to the set of CSFs in the active space. This parametrization ensures the orthonormality of the set of rotated states. We note in passing that using the CSF basis in eq 4 instead of a customized basis of the orthogonal complement, as described in refs (38) and (39) , comes at the cost of a slightly increased number of rotation parameters. Accordingly, this choice introduces a minor redundancy in the set of parameters that needs to be taken care of during the solution of the Λ-equations (cf. eqs 14 and 15).^28,32^ Yet, in this way, a transformation between the customized basis and the CSF basis can be avoided.

The electronic Hamiltonian is used in the second-quantized and spin-free form
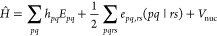
5where *h*_*pq*_ and (*pq*|*rs*) are the one- and two-electron integrals, *V*_nuc_ is the classical nuclear repulsion term, and *E*_*pq*_ and *e*_*pq, rs*_ are the spin-traced one- and two-electron excitation operators.^38^

### Configuration-Based Heatbath CI

**Figure 1:**
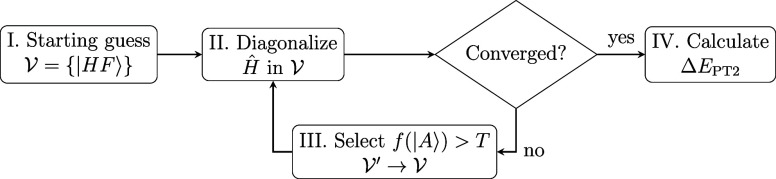
Main algorithmic steps of the HCI method. The steps II and III are iterated over until a convergence criterion is met. In step III, the CFGs outside of  are selected based on an importance function *f* and a threshold *T*.

In a previous work, we reported an orbital-configuration-based heat-bath selected-CI algorithm, CFG-HCI, which we shall hereafter refer to as HCI.^36^ The general scheme of the HCI method is outlined in Figure 1. In the HCI algorithm, the wave function is expanded iteratively until a certain convergence criterion is satisfied. By making the selection threshold *T* smaller, it is possible to converge to FCI accuracy. An important aspect of the present method is the use of orbital configurations as the organizational units of the wave function. That is, the selection, as well as the internal logic of the method, works directly on the CFGs instead of CSFs.^36,40^ The CSFs, which form the many-body basis of the wave function, are implicitly assigned to each CFG. In fact, all possible spin couplings are included for each orbital configuration. The resulting HCI wave function is thereby less compact, since several unimportant CSFs may be included. On the other hand, the method is well-vectorizable and therefore amenable to an efficient computer implementation. The detailed information on the spin couplings is only relevant during the calculation of the electronic coupling coefficients. These quantities are efficiently evaluated using a recursive Kotani-Yamanouchi scheme.^37^ The use of CSFs ensures that the HCI wave function is always an eigenfunction of the  and  operators which becomes particularly beneficial in regions where multiple states of different total spin come close in energy and start to cross.

A central component of the HCI method is the configuration selection scheme; see step III in Figure 1. There are multiple ingredients involved in CFG selection that make it efficient. First, assuming a set of configurations, , a subspace of the so-called generator configurations (generators) is formed:^40,41^
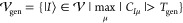
6where *T*_gen_ denotes a user-defined threshold for selecting the generators. The generator configurations are then used for creating the singly and doubly excited candidate CFGs to be selected in the wave function. To avoid creating all of the doubly excited CFGs, we make use of the HCI algorithm developed by Holmes et al.^11^ The HCI selection criteria may be written as follows:
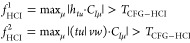
7

Essentially, these criteria stem from the original CIPSI method by discarding the denominator and simplifying the numerator.^11,14,42^ The benefit of discarding the denominator is that the computational scaling is reduced from  to . Another drastic performance improvement comes from the algorithmic details of the HCI method. For that, we refer the reader to the previous works.^11,36^ Here, it shall be summarized that in the HCI method, the quantities *h*_*tu*_, (*tu*|*vw*), and *C*_*I*μ_ are sorted before the selection starts. This allows for an early termination of the loops over orbital indices and configurations in the selection procedure. Furthermore, the doubly excited CFG generation and selection happen simultaneously, while those CFGs that are deemed unimportant are never generated. While its selection procedure is fast, the HCI method is known to overselect determinants for the wave function.^43^ This is due to the discarding of the Epstein–Nesbet perturbation theory denominator, which tends to lower the values of the CIPSI amplitudes. Therefore, we prune the set of selected HCI configurations, _HCI_, further through the subsequent application of the CIPSI selection criterion:
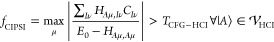
8

Note that the same selection threshold *T*_CFG-HCI_ is used here as that for the HCI selection. The CIPSI-selected CFGs, _CIPSI_, are then appended to the current set of CFGs, , and the Hamiltonian is diagonalized again. The HCI iterations are terminated either when no new CFGs are found or when the energy difference between two subsequent iterations is below a specified threshold (10^–5^ Eh in the current implementation). It is possible to calculate the active space EN-PT2 correction on top of the variation of the HCI energy. However, Smith et al. have found that its contributions to the nuclear gradient of determinant-based HCI for electronic ground states are small,^44^ and thus, we discard them during the computation of nuclear gradients altogether.

We adopted the simple approach by Holmes et al.^45^ for the calculation of electronic excited states with HCI. The CFGs are selected for each state separately, and then, a union is formed and appended to the wave function. In excited-state calculations, the HCI iterations start from the set of singly excited configurations instead of just the Hartree–Fock reference. It should be mentioned that different schemes are possible for selection with multiple electronic states. For example, Kim et al.^35^ used the root-mean-squared (RMS) perturbative ASCI amplitudes over the number of states to select a particular Slater determinant.

### The SA-HCISCF Lagrangian

In both exact and approximate state-averaged (SA-) CASSCF calculations, the orbital and state rotation parameters, κ_*pq*_ and *R*_Ψ*I*_, are optimized to minimize a state-averaged energy, . As a consequence, the energy of a specific state Θ is no longer variational, . This makes the straightforward calculation of the nuclear gradient for a specific state difficult, since the orbital response, , would have to be evaluated. A way to avoid this complication is by employing a variational Lagrangian for the state-specific energy.^46^ Following the original work by Stålring et al.,^28^ we define the SA-CASSCF Lagrangian as
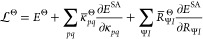
9where  and  denote the Lagrange multipliers for the orbital and CI rotation parameters, respectively. At convergence, the derivatives on the right-hand side vanish, and the Lagrangian  coincides with the energy. This allows for the Lagrange multipliers to be chosen in any desirable way. For the present purposes, a convenient choice is to determine them by requiring the Lagrangian to be variational, i.e.,
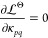
10and
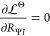
11

With such Lagrange multipliers at hand, the nuclear gradient can be calculated as
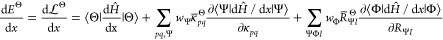
12where only the Hamiltonian has to be differentiated and no orbital response terms arise. The stationary conditions in eq 11 can be written out in more detail as
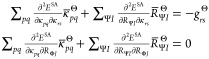
13or in a more concise matrix form:
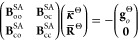
14

Note that the CI gradient on the right-hand side, , is zero since we assume that each electronic state is converged with respect to the CI parameters. Thus, the first step to calculating the nuclear gradient is obtaining the Lagrange multipliers from the Λ-equations in eq 14. We followed previous works and employed the preconditioned conjugate gradient (PCG) algorithm for this task.^28,32,47^ An advantage of the PCG method is that it does not require storing the electronic Hessian **B** in eq 14. Instead, a product of it with the so-called trial vector is required: **σ** = **Bp**. Thus, the elements of the Hessian can be calculated on the fly during the formation of the sigma-vector, **σ**. The explicit terms for the electronic Hessian are given in Appendix A.

Similar to the work of Stålring et al.,^28^ we use the Hessian diagonal as the preconditioner, with the state-averaged states projected out in the CI block. Specifically, the CI part of the preconditioner is given by
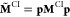
15with
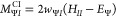
16and
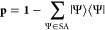
17

This preconditioner can be inverted analytically; for the explicit formulas, we refer to the works of Stålring et al.^28^ or Snyder et al.^32^ The projected preconditioner simplifies the calculation of the σ-vectors as well, because the terms containing CI-vectors can be neglected in calculating the product . For the initial guess, we simply invert the diagonal of the orbital–orbital block in the Hessian (eq 14).

During the solution of the Λ-equations, we make use of the HCI implementation to calculate parts of the Hessian that involve CI derivatives: , , and . For example, for the product , we have to calculate a product between the Hamiltonian and a trial vector,  (see eq 76). Such products are routinely calculated in the CFG-HCI method itself during -diagonalization via the Davidson algorithm.^48,49^ Therefore, we can directly make use of our selected CI code. With a small additional effort, we can make use of the HCI code in calculating  and  as well. Considering next the product, , and the explicit terms in eqs 73–75, we find that the following one- and two-RDMs are to be calculated:
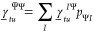
18
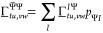
19

The underscore in these terms implies symmetrization as defined in eqs 62 and 63. Since the calculation of the transition RDMs is implemented in our code, these densities can be obtained simply by providing the CI part of the trial vector, **p**_*c*_, as the bra vector to the corresponding function. Finally, the  contribution requires a bit more effort. The strategy is to embed the orbital part of the trial vector into the molecular integrals such that an auxiliary active space Hamiltonian can be formed. This Hamiltonian can then be passed on to a corresponding σ-vector function from the HCI solver. Using the auxiliary Hamiltonian, the contribution to the σ-vector is given by
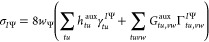
20with the auxiliary molecular integrals defined as^33,38^
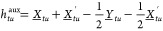
21
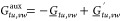
22with the one-electron terms
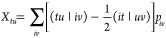
23
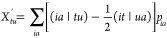
24
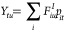
25
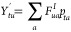
26

The underscore in eqs 21 and 22 means symmetrizations are defined as before. Finally, the two-electron integral terms are given by
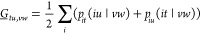
27
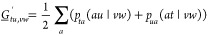
28

In summary, one PCG iteration involves calculations of two HCI σ-vectors and an evaluation of the 1/2-RDMs for each root involved in the state-averaging.

### Analytical Nuclear Gradient

Having obtained the Lagrange multipliers from the Λ-equations, the nuclear gradient may be calculated as^32,33^
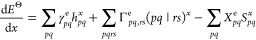
29where , , and  are the geometric derivatives of one-electron, two-electron, and overlap integrals, respectively. In the current implementation, all two-electron integral derivatives (including two- and three-index integral derivatives) are computed utilizing the Libint library in its version 2.8.0.^50^ The Lagrange multipliers are embedded into the effective 1- and 2-RDMs,  and , but also into the connection matrix . Thus, the effective RDMs are given by
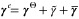
30
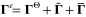
31where the tilde and bar denote the parts containing the orbital and CI Lagrange multipliers, respectively. These contributions to the effective RDMs are given by
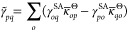
32
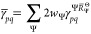
33
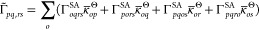
34
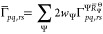
35

Note that in the given CI RDMs, we have assumed that the CI Lagrange multipliers are orthogonal to the CI vectors. Therefore, their corresponding expressions differ slightly compared to Snyder et al.^32^ Analogously, the connection matrix consists of three parts,
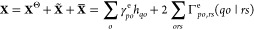
36

The explicit working equations for the effective densities that enter **X̃** and **X̅** are lengthy; therefore, we refer the reader to the work of Snyder et al.^32^ For completeness, we shall also give an expression for the nonadiabatic coupling (NAC) vector
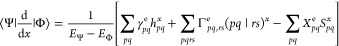
37
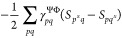
38

In the last term, the *x*-superscript was put above the index of the molecular orbital that is differentiated,
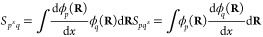
39

In the case of the NAC, the effective RDMs differ compared to the gradient in the first term, which now contains transition densities in place of the state-specific ones,
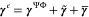
40
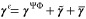
41

The transition densities replace the state-specific densities in the connection matrix, , as well.

### Orbital Optimization

The basis for second-order orbital optimization is the Taylor expansion of the SA-CASSCF energy^38,51−55^
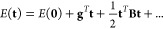
42where **t** denotes the orbital and CI rotation parameters collectively. Truncating the series at the second order, taking a derivative with respect to **t**, and equating it to zero yields the equation for the second-order update of the wave function variables:
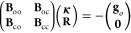
43

Note the close resemblance of this linear system to that in eq 14. The difference is that the electronic gradient and Hessian now correspond to a general CASSCF wave function, which may be either state-specific or state-averaged. As before, the CI gradient is zero, since we assume that the CI variables are obtained from a Davidson diagonalization. It should be mentioned that such a scheme, where the CI and orbital variables are optimized separately, is sometimes referred to as an *alternating scheme*.^53,56^ It is possible to update both sets of variables simultaneously. For example, recently, Helmich-Paris published a method that does so by defining an appropriate orthogonal complement to the CI space spanned by the target states.^39^ Such a scheme might be advantageous in a CASSCF optimization, where the number of CSFs is fixed. However, in the HCI approach, where the size of the CI space can vary, we consider it preferable to build the wave function anew after each orbital optimization step.

It is known that directly solving eq 43 may yield too large orbital steps, **κ**, thereby leading to divergence in the orbital optimization. Therefore, we employ the trust-region augmented Hessian (TRAH) approach that constrains the step length ||***κ***|| to within a trust radius *h*.^39,57,58^ Within the TRAH framework, the orbital update **κ** is obtained by solving the augmented Hessian eigenvalue problem
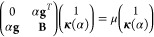
44where μ is the level-shift parameter, and α is the scaling factor. By changing the value of α, it is possible to constrain the orbital step to within the trust radius,
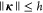
45with
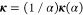
46

The crucial part is obtaining a suitable value for α. For that purpose, we closely followed the work of Høvik et al. and implemented a specific bisection method.^57^ Starting from α_0_ = 1, we increase its value according to α_*k*_ = α_0_·1.5^*k*^ and diagonalize the AH eigenvalue equation, eq 44, until the norm of the step given by eq 46 is inside the trust radius. Based on the last two values of α, the value of α is refined with repeated bisections and AH diagonalizations until the difference between the step length and the trust radius is below a specified threshold. This approach allows for reliable control over the orbital step length. However, more details should be pointed out. Compared to previous works, we do not apply a trust radius control, such as Fletcher’s algorithm.^58−60^ Instead, we use a fixed and fairly conservative default value for the trust radius, *h* = 0.5. In the case of convergence problems, it is left to the user to reduce the value of *h*. It has been reported previously that orbital optimization with SCI wave functions can display oscillatory behavior.^61^ In our tests, this behavior sometimes occurred near the convergence of the orbital optimization. As a remedy, we fix the HCI wave function when the norm of the orbital gradient falls below a specific threshold, which by default is set to 5·10^–4^. If the oscillations occur above this threshold, then the user can change its value accordingly.

At this point, we note that active–active orbital rotations were not considered at all in this work. It is clear that their influence on the total energy decreases with decreasing thresholds to the point of invariance for a complete active space treatment. Several approaches to include active–active orbital rotations in the energy minimization have been published recently.^19,44,56,61^ In our opinion, however, the extra effort required to incorporate such rotations in energy minimizations and gradient calculations is immense and can be circumvented by applying tight thresholds instead. Furthermore, the convergence problems due to the inherent redundancy of the problem will likely also complicate the solution of the Λ equations.

### Density Fitting

Density fitting (DF) allows for the decomposition of four-center electron repulsion integrals (ERIs) in terms of two- and three-center ERIs,^62−67^
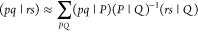
47

A major performance benefit with DF stems from the fact that the two- and three-center integrals are considerably cheaper to compute than the four-center ones. Furthermore, the quantities in eq 47 can be made use of in a highly vectorized manner^65^ in constructing lower-rank intermediates. We applied the DF approximation in the calculation of the SA-HCISCF (and SA-CASSCF) nuclear gradients and NAC vectors, as well as in the calculation of the Lagrange multipliers.

The most time-consuming part of solving the Λ-equations is the calculation of the σ-vector, **σ** = **B**^SA^**p**. Specifically, the contraction of the SA-HCISCF Hessian with the trial vector involves two-electron integrals in the MO basis. One may state that the contraction involves general Coulomb and exchange-like terms, i.e.,
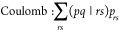
48
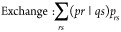
49

The DF approach can be applied to such contractions as follows. First, we can decompose the Coulomb metric over auxiliary basis functions,^65,67^
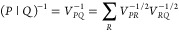
50

This composition always exists since the *V*_*PQ*_ matrix is positive definite.^68^ Multiplying the inverse square root vectors into the three-center ERIs yields
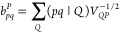
51

Therefore, we may write eq 47 as
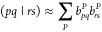
52which bears a close resemblance to the well-known Cholesky decomposition representation of the four-center ERI tensor.^68−70^ Using this factorization, the Coulomb and exchange terms in the σ-vector calculation may be contracted as follows:
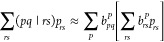
53
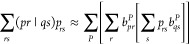
54

The calculation of the first Coulomb term can be done at the cost of  by first calculating a one-index intermediate within the square brackets. Unfortunately, the exchange term still possesses a  scaling with the number of electrons because no lower-rank intermediates can be formed. Nevertheless, the required operations can be carried out efficiently using BLAS level-3 matrix multiplications. In our implementation, the *b*-coefficients are first calculated and stored in the atomic orbital basis. We calculate them only once, before the CASSCF iterations commence. The *b*-coefficients are stored either in memory or on disk, depending on the size of the problem versus the available amount of memory. The transformation of **B**^P^ to the MO basis happens on the fly in the σ-vector calculation. This transformation scales generally as  and is carried out using fast matrix multiplications.

Using density fitting, the geometric derivative of the two-electron integrals can be expressed as^29,30^
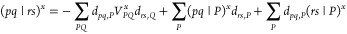
55with the density-fitting coefficients defined as
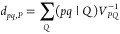
56

Contracting eq 55 with the effective SA-CASSCF 2RDM, eq 31, yields^30^
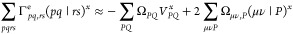
57where the two- and three-index intermediates were introduced:
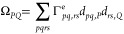
58
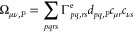
59

All other contractions of derivative integrals can be factorized in an analogous fashion (see Appendix B). In the current implementation, we calculated the Ω-intermediates before evaluating the nuclear derivatives of *V*_*PQ*_ and (μν|*P*). The latter three-index intermediates are first calculated in the MO basis and then transformed to the AO basis on the fly during calculating and contracting with (μν|*P*)^*x*^. For the explicit expressions, we refer the reader to Appendix B. It is apparent that the most expensive intermediates  and  scale approximately as  in computational effort. The latter intermediate scales  in storage, but since one of the indices is internal, its practical storage requirement is typically not prohibitive. In fact, we keep it in memory along with all of the other Ω-intermediates. As a last remark, the presented method is efficient for small- to medium-sized molecules, in our opinion. For very large molecules, however, the presented DF approach becomes expensive in both time and storage due to the quartic and cubic scaling, respectively. In that regime, other approaches, such as the chain-of-spheres exchange (COSX) or tensor-hyper contraction (THC), would likely be more suitable.^71−74^

## Results

**Figure 2:**
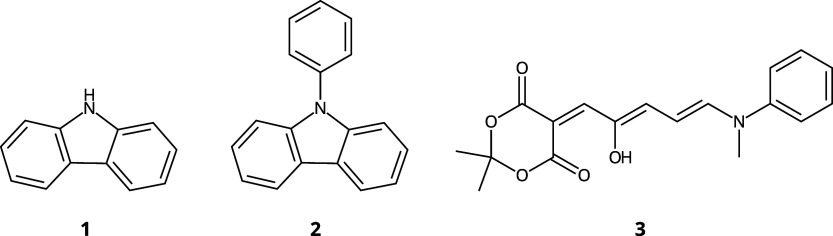
Conjugated systems used in geometry optimization.

**Figure 3:**
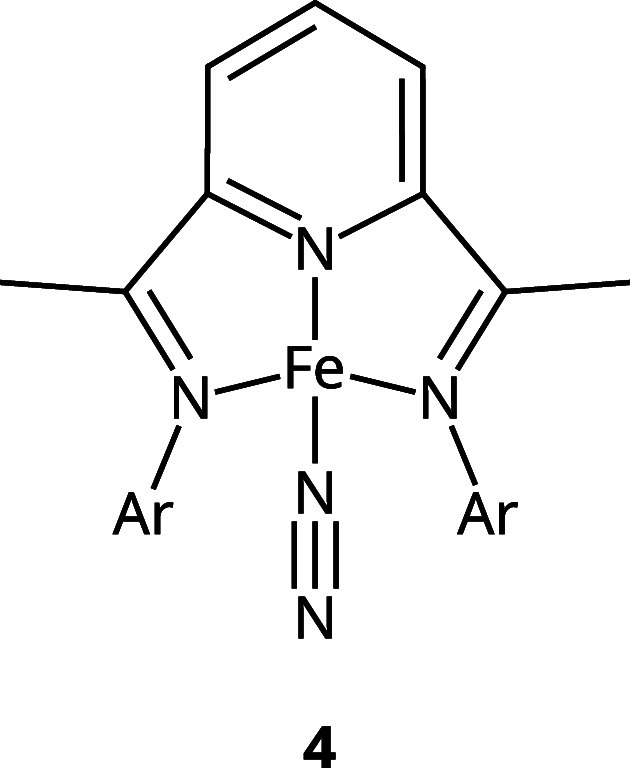
Transition metal catalyst Fe(PDI) with Ar = 2,6-dimethylphenyl. PDI = 2,6-bis[1-(2,6-dimethylphenyl-imino)ethyl]pyridine.

The implemented analytical nuclear gradients were used in geometry optimizations of three conjugated organic compounds and a transition metal complex. The conjugated systems are shown in Figure 2, whereas the transition metal complex Fe(PDI) (**4**) is depicted in Figure 3. Compound **1** (carbazole) is the basis for thermally activated delayed fluorescence (TADF) molecules such as A2B (PhCz),^35,75,76^ while compound **3** is a donor–acceptor Stenhouse adduct.^77^

### Conjugated Organic Compounds

#### Comparison with FCI

In this section, we consider geometry optimizations for electronic ground states and first excited states with varying spin multiplicities of compounds **1** and **3** utilizing HCI and compare them to results obtained with conventional, FCI-based CASSCF. Four different sets of selection thresholds were chosen during the optimizations with HCI as the CI solver, namely (*T*_gen_, *T*_HCI_): *T*_1_ = (10^–2^, 10^–4^), *T*_2_ = (10^–2^, 10^–5^), *T*_3_ = (10^–2^, 10^–6^), and *T* = (10^–3^, 10^–5^). The thresholds were tightened sequentially by using the geometry with a previous threshold as the starting guess. We believe that starting with more loose (larger) thresholds allows for an efficient yield of better starting geometries. Then, more accurate structures can be obtained in fewer iterations by using tighter thresholds. The optimizations were carried out for different active space sizes, varying between (12e, 12o) and (16e, 16o). A summary of differences in final energies of the ground and first excited states, Δ*E* = *E*_HCI_ - *E*_FCI_, root-mean-square deviations of all chemical bonds and bond angles, as well as the fraction of selected CSFs for all combinations of active spaces and selection thresholds, is presented in Table 1. Electronic states are labeled according to their spin multiplicity (*S*, *D*, and *T*) and energetic order (0 = ground state and 1 = first excited state). As expected, with decreasing (tighter) thresholds, the errors with respect to the FCI diminish. Interestingly, the differences between *T*_2_ and *T*_3_ seem negligible. In particular, the CI expansion lengths stay roughly the same except for the last result on the C molecule. Thus, in order to improve accuracy, it appears more beneficial to tighten the generator thresholds, *T*_gen_. In most cases, except for the pair of *D* states of compound **1**, the energy differences between FCI and HCI are larger for the first excited state than for the ground state (cf. columns 2 and 3 of Table 1). Although the errors in the energies are sizable, the differences in bond distances and angles are much less significant. In the case of the calculation of the *S*_1_ state of **2** with a (16e, 16o) active space, the errors with default thresholds are 0.0014 Å and 0.0469 degrees for the bond lengths and angles, respectively.

**Table 1:** Comparison of HCI Geometries Utilizing Different Sets of Selection Thresholds (See Text) Vs Results from FCI Calculations^a^

*T*	Δ*E*_0_	Δ*E*_1_	RMSD (bond)	RMSD (angle)	% (CSFs)
**Compound 1**					
***S***_**1**_**(12e,12o)**					
1	0.6	2.3	0.0006	0.0141	12.17
2	0.4	1.4	0.0004	0.0091	17.56
3	0.4	1.4	0.0004	0.0088	18.24
4	0.0	0.1	0.0000	0.0023	39.18
***D***_**0**_**(11e, 12o)**					
1	1.6	1.5	0.0005	0.0164	15.68
2	1.5	1.4	0.0005	0.0154	16.90
3	1.5	1.3	0.0005	0.0154	16.93
4	0.0	0.0	0.0000	0.0023	68.06
***T***_**1**_**(12e, 12o)**					
1	1.2	1.7	0.0006	0.0242	23.63
2	0.7	1.3	0.0004	0.0175	30.96
3	0.7	1.3	0.0004	0.0172	31.32
4	0.0	0.1	0.0000	0.0036	75.28
***S***_**1**_**(14e, 13o)**					
1	0.8	2.8	0.0007	0.0163	5.99
2	0.6	1.8	0.0004	0.0131	9.78
3	0.6	1.7	0.0004	0.0121	11.42
4	0.0	0.1	0.0000	0.0023	27.08
***D***_**0**_**(13e, 13o)**					
1	2.8	2.2	0.0008	0.0166	7.86
2	2.6	2.0	0.0007	0.0144	9.02
3	2.6	2.0	0.0007	0.0144	9.05
4	0.1	0.1	0.0001	0.0036	46.66
***T***_**1**_**(14e, 13o)**					
1	1.6	2.2	0.0007	0.0336	10.60
2	0.9	1.4	0.0005	0.0206	17.91
3	0.9	1.4	0.0005	0.0206	18.23
4	0.1	0.1	0.0000	0.0000	45.47
**Compound 3**					
***S***_**1**_**(16e, 16o)**					
1	1.5	6.9	0.0022	0.0711	0.45
2	0.8	4.1	0.0014	0.0469	1.30
3	0.8	3.9	0.0014	0.0466	1.73
4	0.2	0.7	0.0002	0.0159	4.63

a The energy differences are given in millihartrees (mHa), and the RMSD values are given in Å and degrees for bond lengths and bond angles, respectively. The last column contains the fraction of selected CSFs in % compared to the FCI wave function.

For all optimizations presented in this section, the timings were dominated by the CI steps. Yet, for all cases, except the ones on the *S*_1_ state of **2** with a (16e, 16o) active space, it is advisable to use FCI instead of HCI, since its efficiency due to a highly vectorized implementation leads to shorter calculation times. For the largest calculation of (16e, 16o), it is more beneficial to use the selected CI method. For example, when *T*_4_ thresholds were used, the calculation took 2 days and 1 h (17 optimization cycles), whereas the FCI calculation took 4 days and 16 h (13 optimization cycles). With default selection thresholds, the optimization time was reduced to 6 and a half h (12 optimization cycles) on a single computing node without any significant negative effects on the RMSD of bond angles and distances (see Table 1).

#### Large Active Spaces

In this section, we proceed with discussing geometry optimizations that employ active spaces beyond the FCI limits. More precisely, we utilized active space sizes of (18e, 18o) and (20e, 19o) during optimizations of **2**, both in singlet and triplet excited states. Moreover, we optimized **3** with an active space of (18e, 17o). With active spaces of this magnitude, FCI calculations are at the edge of feasibility and require intense computational efforts, even without geometry optimizations. Therefore, the reference geometries in this section were taken from HCI calculations with very tight thresholds, *T*_gen_ = 10^–3^ and *T*_var._ = 10^–6^, instead. The reference wave function dimensions and timings are listed in Table 3. In the case of the first excited triplet state of **2** with an (20e, 19o) active space, the optimization took around 21 and a half days, which is prohibitively costly. However, it was carried out only to obtain a reference geometry for the present comparison purposes. Final energy differences, Δ*E* = *E*_HCI_ – *E*_HCI(tight)_, and calculated RMSDs of geometrical parameters with respect to the aforementioned references for different sets of thresholds are given in Table 2. These results confirm the observations made in the previous section. Optimizations conducted with default thresholds, *T*_gen_ = 10^–2^ and *T*_var._ = 10^–5^, yield geometrical parameters that are sufficiently close to the reference geometries. In contrast to the cases discussed previously, the time savings are substantial: No optimization took longer than 14 h. At this point, we would like to mention that all optimizations presented in this section with selection thresholds *T*_*n*_ used the optimized geometry with *T*_*n*–1_ as the starting geometry. On the one hand, this explains why occasionally optimizations with *T*_3_ took less time than those with *T*_2_ thresholds. On the other hand, this emphasizes our assessment that tightening the thresholds beyond the default setting leads to significantly higher computational cost while not offering considerably more accurate results. If, however, accuracy beyond what the default thresholds offer is necessary, we strongly recommend to follow this procedure of preoptimizing with loose thresholds and only perform the last optimization cycles with tight selection thresholds. This will result in substantial computational savings with (probably) no loss of accuracy (Table 3).

**Table 2:** Comparison of HCI Geometries of **2** with Different Thresholds (Defined in the Text) versus Results from Calculations with Very Tight Thresholds^a^

*T*	Δ*E*_0_	Δ*E*_1_	RMSD (bond)	RMSD (angle)	Time [h]^b^
**Compound 2**					
***S***_**1**_**(18e, 18o)**					
1	8.0	16.2	0.0019	0.0692	1
2	5.1	9.9	0.0014	0.0396	4
3	4.9	9.4	0.0013	0.0391	2
4	0.8	1.8	0.0003	0.0192	25
***T***_**1**_**(18e, 18o)**					
1	33.1	21.1	0.0063	0.1826	7
2	7.7	9.5	0.0019	0.0573	47
3	6.0	7.3	0.0012	0.0427	12
4	1.5	2.0	0.0006	0.0163	108
***S***_**1**_**(20e, 19o)**					
1	8.7	17.7	0.0020	0.0767	2
2	5.2	10.5	0.0014	0.0455	4
3	5.1	9.9	0.0013	0.0442	4
4	1.0	2.2	0.0004	0.0256	25
***T***_**1**_**(20e, 19o)**					
1	15.5	20.4	0.0034	0.1489	6
2	7.7	9.5	0.0016	0.0645	14
3	6.6	7.8	0.0013	0.0493	20
4	1.9	2.3	0.0004	0.0178	84
**Compound 3**					
***S***_**1**_**(18e, 17o)**					
1	2.1	8.0	0.0025	0.0839	3
2	1.3	4.5	0.0013	0.0457	3
3	1.3	4.2	0.0013	0.0443	5
4	0.2	0.7	0.0002	0.0083	16

a The energy differences are given in mHa, and the RMSD values are given in Å and degrees for bond lengths and bond angles, respectively.

b 3 nodes with AMD EPYC 7451 CPUs and 503GB RAM were used. 48 OMP threads were launched on each node.

**Table 3:** Numbers of CSFs in the Final Wave Function, Numbers of Geometry Optimization Steps, and Total Wall Times in Hours

	Compound 3	Compound 2			
	*S*_1_(18e, 17o)	*S*_1_(18e, 18o)	*T*_1_(18e, 18o)	*S*_1_(20e, 19o)	*T*_1_(20e, 19o)
***N***(CSF)	6633549	20838300	37732868	31629317	59199064
***N***(iter)	4	7	5	6	6
**Time [h]**^a^	19	159	312	199	514

a 3 nodes with AMD EPYC 7451 CPUs and 503 GB RAM were used. 48 OMP threads were launched on each node.

### Fe(PDI)

To further demonstrate the capabilities of the current implementation, we optimized the geometry of the first excited triplet state of the Fe(PDI) complex **4** (see Figure 3). It needs to be stressed at this point that excited states with a specific total spin can only be directly targeted by the reported method because the underlying HCI is fully spin-adapted. If spin purity is not enforced, one would first have to identify the targeted state through a multiroot calculation and would further run the risk of switching spin states in the course of the geometry optimization.

**Figure 4:**
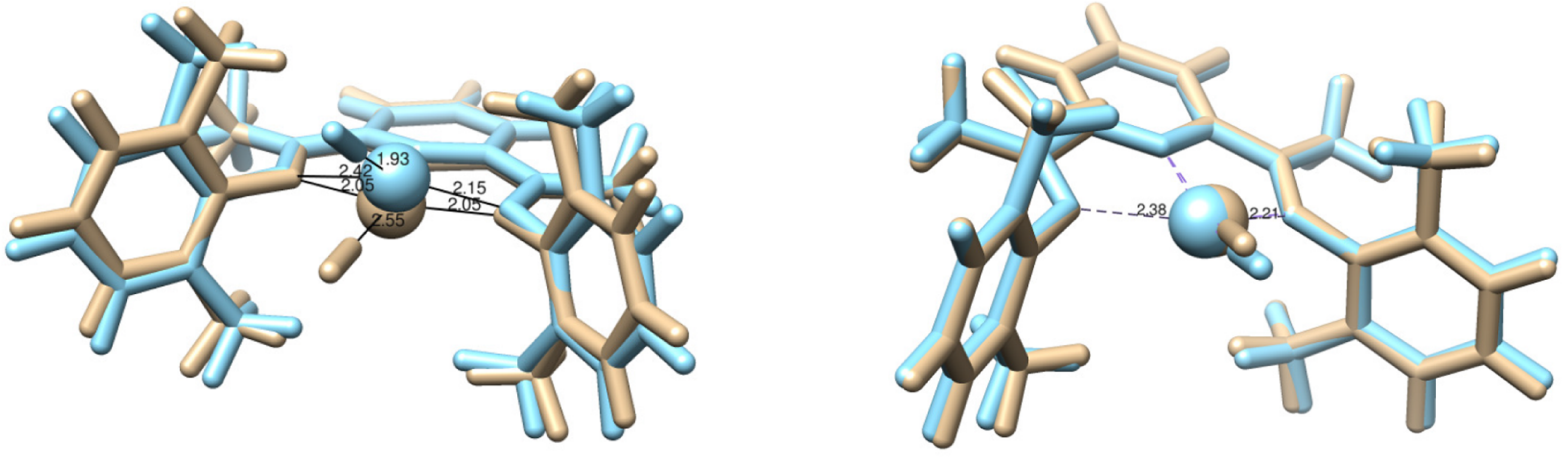
Overlay of Fe(PDI) geometries for the first excited triplet state obtained with (24e, 23o) (left) and (30e, 30o) (right) active spaces, respectively. The geometries obtained with def2-SVP and def2-TZVP basis sets are colored beige and magenta, respectively.

Separate optimizations were conducted with the def2-SVP and def2-TZVP basis sets for two different active spaces, (24e, 23o) and (30e, 30o). The former choice was made based on considerations using the ASS1ST tool.^78,79^ Calculations with the def2-TZVP basis set used the same active space sizes, but the starting orbitals were taken from a preceding HCISCF calculation with a (40e, 40o) active space. The large active space of (30e, 30o) was taken liberally without a specific selection of orbitals. An overlay of all optimized geometries is shown in Figure 4. Notably, the final geometries differ more between the basis sets for the smaller active space. For example, when the def2-TZVP basis set is utilized, the Fe atom and the N_2_ ligand move out of the plane, while this is not observed for the def2-SVP basis set or any of the optimizations with the larger active space.

The corresponding calculation times, together with computed excitation energies, the number of required geometry optimization cycles, the number of selected CSFs in the final iteration, and total wall clock times, are given in Table 4. The numbers of iterations required to converge the optimizations are modest. However, the (30e, 30o) active space calculations took a significant amount of time. Naturally, a larger active space increases computational costs due to the  scaling in the HCI method. Furthermore, the wave functions consist of more CSFs compared to the (24e, 23o) active space. It was evident that the Davidson diagonalization in the CI solver is the time bottleneck in these calculations. We consider our diagonalization method efficient due to the algorithmic details. Nevertheless, there is some room for improvement. For example, the wave function could be made more compact by considering active–active orbital rotations during energy minimization. Furthermore, fewer CSFs would likely be selected by using averaged EN-PT1 amplitudes instead of forming the union of selected CFGs for each electronic state. It might also be possible to enhance the Davidson diagonalization itself by storing the Hamiltonian as a sparse matrix beforehand. This approach can be expected to be most suitable when the CSFs are selected individually, as, for example, in the iCI and CSF-ICE methods.

**Table 4:** Energy Differences between First Two Triplet States of **4**, Number of CSFs in the Final Iteration, Number of Geometry Optimization Iterations, and Total Wall Time in Hours

Calculation	Δ*E* [eV]	*N*(CSF)N(CSF)	*N*(iter)N(iter)	*T* [h]T[h]^a^
(24e, 23o)/def2-SVP	0.17	3806351	14	59
(30e, 30o)/def2-SVP	0.21	22323423	13	582
(24e, 23o)/def2-TZVP	0.15	8156892	11	86
(30e, 30o)/def2-TZVP	0.10	18244504	11	262

a 3 nodes with AMD EPYC 7451 CPUs and 503GB RAM were used. 48 OMP threads were launched on each node.

## Computational Details

The DASA molecule, **3** in Figure 2, was selected from the work by Noirbent et al.^77^ The carbazole and PhCz molecules, **1** and **2** in Figure 2, were taken from a recent study by Kim et al.^35^ Before carrying out the SA-CASSCF and SA-HCISCF geometry optimizations, the ground states of the A, B, and C molecules were preoptimized using the conformer–rotamer ensemble sampling tool (CREST).^80,81^ The starting geometry of the triplet state Fe(PDI) (**4** in Figure 3) was taken from a study by Smith et al.^44^ No preliminary CREST optimizations were done for D.

In order to commence with the geometry optimizations, FCI-ASS1ST^78,79^ calculations were carried out to yield the appropriate active space sizes and starting orbitals in most cases. For that purpose, the ASS1ST methodology was employed by considering both the ground and the first excited state in a state-averaged manner.^79^ In this work, we did not use the IPEA-shift approximation^79^ that avoids the calculation of the expensive (A16)/(A22)^82^ terms. No strict natural orbital occupation number thresholds were applied for selecting the active space sizes. Instead, the inclusion of additional orbitals into the active space was judged based on the extent to which the occupations of specific orbitals differ relative to the rest of the internal/virtual pseudonatural orbitals. By visually inspecting the plotted occupation numbers, it is intuitive and relatively straightforward to select the active spaces. The selected active space was further scrutinized by performing a CASSCF calculation. If none of the resulting active orbitals had insignificant occupation numbers (close to 0 or 2), then the active space was kept. Only in the case of Fe(PDI) calculations with the def2-TZVP basis set, no ASS1ST procedure was used. There, a large HCISCF (40e, 40o) calculation was carried out in order to obtain the initial orbitals. Smaller active space calculations were then started from these orbitals.

We used our Humboldt-Multireference (HUMMR) program^36,37^ (formerly named MOLBLOCK)^83^ for calculating the SA-CASSCF and SA-HCISCF nuclear gradients. The calculated gradients were used for performing geometry optimizations with the ORCA program package. Specifically, ORCA5.0.3^84,85^ was used in geometry optimizations by invoking HUMMR as an external program. By following the steps described in the ORCA manual, it was straightforward to conduct a geometry optimization, where ORCA acts solely as a geometry optimizer, and the electronic structure part and nuclear gradient calculation are handled by HUMMR. Tight convergence criteria were used in all of the geometry optimizations. The final structures were compared by calculating the root-mean-square deviation (RMSD) between the bond lengths and the bond angles.

Up to three computer nodes with 3 corresponding MPI processes were used in the geometry optimizations. We used 24 or 48 OpenMP threads per node. The gradient was calculated with both FCI and HCI as the active space CI solver. In all of the HUMMR calculations, we used the fully CI-coupled FNR approach in HUMMR for optimizing the orbitals. The def2-SVP basis sets were used in most of the calculations, with exceptions being molecules **1** and **4,** for which cc-pVDZ^86^ and def2-TZVP^87^ were used, respectively. The universal def2-JK auxiliary basis set was employed in the density fitting for two-electron integrals.^88^ Neither active–active orbital rotations nor the PT2 correction were considered in any HCISCF or nuclear gradient calculations.

## Conclusions

In this work, we reported on the implementation of analytical nuclear gradients with exact (FCI) and approximate (CFG-HCI) SA-CASSCF wave functions. By using our previously implemented HCI algorithm, it was possible to tackle active spaces larger than those with FCI. A pertinent feature of our HCI method is that it is spin-adapted. This makes studying potential energy surfaces with different multiplicities reliable because converging to the correct spin state is guaranteed. Aside from the analytical nuclear gradient, we also implemented the calculation of the nonadiabatic coupling vector. This quantity could be utilized in locating conical intersections.^89,90^ At the time of this work, we did not have access to an appropriate geometry optimizer, unfortunately. Thus, locating conical intersections could be one of the main applications of the implemented methods. Compared to some previous works, we did not incorporate the PT2 correction in formulating the SA-HCISCF Lagrangian. The close agreement between our results obtained from HCI and FCI-based geometry optimizations confirmed the findings of Smith et al. that, with reasonably tight selection thresholds, the influence of these terms is comparably small.^44^

Developing the required infrastructure for calculating the gradients yielded a fully CI-coupled (FNR) second-order orbital optimization method. The FNR method avoids the common pitfalls of first-order approaches, such as trailing convergence or converging to saddle points. This method can be used together with the aforementioned HCI wave functions, thereby enabling approximate CASSCF calculations with up to 40 active orbitals. It is clear that the FNR approach requires more computational effort in one orbital optimization macroiteration than a typical alternative optimizer would. However, the reliability and the reduction in the number of macroiterations can render the use of FNR preferable, even more so for large active space calculations, where the CI step is rate-determining.

We also attempted to tackle the challenge of treating molecules with a larger atomic orbital dimension. Employing the density fitting approximation allows the presented methods to be efficiently used on medium-sized molecules. Yet, eventually, the inherent scaling of the density fitting methodology becomes prohibitive. As discussed earlier, at least two approaches exist that enable further reduction of the computational scaling: seminumerical exchange and tensor hypercontraction.^71−74^ Investigating these approaches is a worthwhile effort left for the future. Although several challenges remain in this work, we hope that our effort constitutes a stepping stone toward predicting both ground and excited state potential energy surfaces accurately and efficiently.

## Data Availability

The presented code can be downloaded free of charge at https://www.chemie.hu-berlin/.de/en/forschung-en/theoretical-chemistry/downloads. The structures of all compounds discussed in this work, alongside the orbital coefficients of all molecular orbitals (in human-readable form and HUMMR input format) underlying the presented calculations, are provided in the Supporting Information.

## Appendix

### SA-CASSCF Electronic Hessian

The core, active and generalized Fock matrices are given by^51^

60

where *γ*_*pq*_ and Γ_*pq, rs*_ are the one- and two-electron RDMs, respectively. The orbital gradient is calculated from the generalized Fockian as

61

The generalized and symmetrized 1/2-RDMs we define as

62

63

where *X* and *Y* denote arbitrary vectors in the Fock space.

#### Orbital-orbital derivatives

The general expression for the orbital–orbital part of the CASSCF Hessian is given by

64

Below are given the six unique parts of the orbital–orbital block of the CASSCF Hessian.^51,91^

65

66

67

68

69

70

In the above expressions, we assumed the usual symmetries of the two-electron integrals and that the core and active Fockians are symmetric. The latter is symmetric since the 1RDM (not transition-), γ_*pq*_, is symmetric. Note that the very last term in the expression for *B*_*ta, ub*_ differs from Siegbahn et al.^51^ They assumed a CAS wave function, for which the active–active orbital gradient is zero

71

and hence, the generalized Fockian is symmetric. This condition does not hold generally for the SCI wave functions that are used in this work. Additionally, we have included the missing orbital gradient contributions for *B*_*it, ja*_, *B*_*it, ua*_ and *B*_*ia, tb*_. Assuming a converged (approximate) CASSCF wave function, these terms vanish.

#### Orbital-CI derivatives

The orbital-CI CASSCF derivative is given by

72

Below are given specific parts of the orbital-CI block of the CASSCF Hessian. The CI-orbital block is equivalent due to symmetry. The terms in bold font do not have to be evaluated when the orthogonal preconditioner described in Section E is employed.

73

74

75

#### CI–CI derivatives

The CI–CI part of the CASSCF Hessian is given by

76

where the bold font terms vanish for the orthogonal preconditioner.

### Two-electron integral derivative intermediates with density fitting

The two-electron contributions to the SA-CASSCF nuclear gradient are given by

77

We shall next write out the state-specific orbital and CI contributions under the density-fitting approximation. Omitting the lengthy but straightforward derivations, the resulting terms are given by

78

79

80

In the above expressions, few additional quantities were defined:

81

82

Finally, the Ω-intermediates are defined in eqs (B7) to (B17).

83

84

85

86

87

88

89

90

91

92

93

## Supporting Information Available

Plots of internal and external pseudonatural orbital occupation numbers obtained with ASS1ST (PDF)

## References

[ref1] Khedkar A.; Roemelt M. Modern multireference methods and their application in transition metal chemistry. Phys. Chem. Chem. Phys. 2021, 23, 17097–17112. 10.1039/D1CP02640B. 34355719

[ref2] Lischka H.; Nachtigallová D.; Aquino A. J. A.; Szalay P. G.; Plasser F.; Machado F. B. C.; Barbatti M. Multireference Approaches for Excited States of Molecules. Chem. Rev. 2018, 118, 7293–7361. 10.1021/acs.chemrev.8b00244. 30040389

[ref3] Gaggioli C. A.; Stoneburner S. J.; Cramer C. J.; Gagliardi L. Beyond density functional theory: the multiconfigurational approach to model heterogeneous catalysis. ACS Catal. 2019, 9, 8481–8502. 10.1021/acscatal.9b01775.

[ref4] Chan G. K.-L.; Head-Gordon M. Highly correlated calculations with a polynomial cost algorithm: A study of the density matrix renormalization group. J. Chem. Phys. 2002, 116, 4462–4476. 10.1063/1.1449459.

[ref5] Sharma S.; Chan G. K.-L. Spin-adapted density matrix renormalization group algorithms for quantum chemistry. J. Chem. Phys. 2012, 136 (12), 124121 10.1063/1.3695642. 22462849

[ref6] Keller S.; Reiher M. Spin-adapted matrix product states and operators. J. Chem. Phys. 2016, 144 (13), 134101 10.1063/1.4944921. 27059556

[ref7] Petruzielo F.; Holmes A.; Changlani H. J.; Nightingale M.; Umrigar C. Semistochastic projector monte carlo method. Phys. Rev. Lett. 2012, 109, 230201 10.1103/PhysRevLett.109.230201. 23368167

[ref8] Blunt N. S.; Smart S. D.; Kersten J. A. F.; Spencer J. S.; Booth G. H.; Alavi A. Semi-stochastic full configuration interaction quantum Monte Carlo: Developments and application. J. Chem. Phys. 2015, 142, 184107 10.1063/1.4920975. 25978883

[ref9] Dobrautz W.; Smart S. D.; Alavi A. Efficient formulation of full configuration interaction quantum Monte Carlo in a spin eigenbasis via the graphical unitary group approach. J. Chem. Phys. 2019, 151 (9), 094104 10.1063/1.5108908. 31492066

[ref10] Giner E.; Scemama A.; Caffarel M. Using perturbatively selected configuration interaction in quantum Monte Carlo calculations. Can. J. Chem. 2013, 91, 879–885. 10.1139/cjc-2013-0017.

[ref11] Holmes A. A.; Tubman N. M.; Umrigar C. Heat-bath configuration interaction: An efficient selected configuration interaction algorithm inspired by heat-bath sampling. J. Chem. Theory Comput. 2016, 12, 3674–3680. 10.1021/acs.jctc.6b00407. 27428771

[ref12] Liu W.; Hoffmann M. R. iCI: Iterative CI toward full CI. J. Chem. Theory Comput. 2016, 12, 1169–1178. 10.1021/acs.jctc.5b01099. 26765279

[ref13] Tubman N. M.; Lee J.; Takeshita T. Y.; Head-Gordon M.; Whaley K. B. A deterministic alternative to the full configuration interaction quantum Monte Carlo method. J. Chem. Phys. 2016, 145 (4), 044112 10.1063/1.4955109. 27475353

[ref14] Garniron Y.; Applencourt T.; Gasperich K.; Benali A.; Ferté A.; Paquier J.; Pradines B.; Assaraf R.; Reinhardt P.; Toulouse J.; et al. Quantum package 2.0: An open-source determinant-driven suite of programs. J. Chem. Theory Comput. 2019, 15, 3591–3609. 10.1021/acs.jctc.9b00176. 31082265

[ref15] Prentice A. W.; Coe J. P.; Paterson M. J. Modular approach to selected configuration interaction in an arbitrary spin basis: Implementation and comparison of approaches. J. Chem. Theory Comput. 2023, 19, 9161–9176. 10.1021/acs.jctc.3c00897. 38061390 PMC10753805

[ref16] Ghosh D.; Hachmann J.; Yanai T.; Chan G. K.-L. Orbital optimization in the density matrix renormalization group, with applications to polyenes and β-carotene. J. Chem. Phys. 2008, 128 (14), 144117 10.1063/1.2883976. 18412433

[ref17] Ma Y.; Knecht S.; Keller S.; Reiher M. Second-order self-consistent-field density-matrix renormalization group. J. Chem. Theory Comput. 2017, 13, 2533–2549. 10.1021/acs.jctc.6b01118. 28485978

[ref18] Levine D. S.; Hait D.; Tubman N. M.; Lehtola S.; Whaley K. B.; Head-Gordon M. CASSCF with extremely large active spaces using the adaptive sampling configuration interaction method. J. Chem. Theory Comput. 2020, 16, 2340–2354. 10.1021/acs.jctc.9b01255. 32109055

[ref19] Guo Y.; Zhang N.; Lei Y.; Liu W. iCISCF: an iterative configuration interaction-based multiconfigurational self-consistent field theory for large active spaces. J. Chem. Theory Comput. 2021, 17, 7545–7561. 10.1021/acs.jctc.1c00781. 34757746

[ref20] Dobrautz W.; Weser O.; Bogdanov N. A.; Alavi A.; Li Manni G. Spin-pure stochastic-CASSCF via GUGA-FCIQMC applied to iron–sulfur clusters. J. Chem. Theory Comput. 2021, 17, 5684–5703. 10.1021/acs.jctc.1c00589. 34469685 PMC8444347

[ref21] Guo S.; Watson M. A.; Hu W.; Sun Q.; Chan G. K.-L. N-electron valence state perturbation theory based on a density matrix renormalization group reference function, with applications to the chromium dimer and a trimer model of poly (p-phenylenevinylene). J. Chem. Theory Comput. 2016, 12, 1583–1591. 10.1021/acs.jctc.5b01225. 26914415

[ref22] Freitag L.; Knecht S.; Angeli C.; Reiher M. Multireference perturbation theory with cholesky decomposition for the density matrix renormalization group. J. Chem. Theory Comput. 2017, 13, 451–459. 10.1021/acs.jctc.6b00778. 28094988 PMC5312874

[ref23] Song Y.; Cheng Y.; Ma Y.; Ma H. Multi-reference Epstein–Nesbet perturbation theory with density matrix renormalization group reference wavefunction. Electron. Struct. 2020, 2, 014002 10.1088/2516-1075/ab72db.

[ref24] Guo Y.; Sivalingam K.; Kollmar C.; Neese F. Approximations of density matrices in N-electron valence state second-order perturbation theory (NEVPT2). II. The full rank NEVPT2 (FR-NEVPT2) formulation. J. Chem. Phys. 2021, 154 (21), 214113 10.1063/5.0051218. 34240984

[ref25] Park J. W. Dynamic Correlation on the Adaptive Sampling Configuration Interaction (ASCI) Reference Function: ASCI-DSRG-MRPT2. J. Chem. Theory Comput. 2023, 19, 6263–6272. 10.1021/acs.jctc.3c00688. 37611192

[ref26] Choudhury A.; Santra S.; Ghosh D. Understanding the Photoprocesses in Biological Systems: Need for Accurate Multireference Treatment. J. Chem. Theory Comput. 2024, 20 (12), 4951–4964. 10.1021/acs.jctc.4c00027. 38864715

[ref27] Tran L. N.; Shea J. A.; Neuscamman E. Tracking excited states in wave function optimization using density matrices and variational principles. J. Chem. Theory Comput. 2019, 15, 4790–4803. 10.1021/acs.jctc.9b00351. 31393725

[ref28] Stålring J.; Bernhardsson A.; Lindh R. Analytical gradients of a state average MCSCF state and a state average diagnostic. Mol. Phys. 2001, 99, 103–114. 10.1080/002689700110005642.

[ref29] Delcey M. G.; Freitag L.; Pedersen T. B.; Aquilante F.; Lindh R.; González L. Analytical gradients of complete active space self-consistent field energies using Cholesky decomposition: Geometry optimization and spin-state energetics of a ruthenium nitrosyl complex. J. Chem. Phys. 2014, 140 (17), 174103 10.1063/1.4873349. 24811621

[ref30] Delcey M. G.; Pedersen T. B.; Aquilante F.; Lindh R. Analytical gradients of the state-average complete active space self-consistent field method with density fitting. J. Chem. Phys. 2015, 143 (4), 044110 10.1063/1.4927228. 26233110

[ref31] Snyder J. W. Jr; Hohenstein E. G.; Luehr N.; Martínez T. J. An atomic orbital-based formulation of analytical gradients and nonadiabatic coupling vector elements for the state-averaged complete active space self-consistent field method on graphical processing units. J. Chem. Phys. 2015, 143 (15), 154107 10.1063/1.4932613. 26493897

[ref32] Snyder J. W. Jr; Fales B. S.; Hohenstein E. G.; Levine B. G.; Martínez T. J. A direct-compatible formulation of the coupled perturbed complete active space self-consistent field equations on graphical processing units. J. Chem. Phys. 2017, 146 (17), 174113 10.1063/1.4979844. 28477593

[ref33] Freitag L.; Ma Y.; Baiardi A.; Knecht S.; Reiher M. Approximate analytical gradients and nonadiabatic couplings for the state-average density matrix renormalization group self-consistent-field method. J. Chem. Theory Comput. 2019, 15, 6724–6737. 10.1021/acs.jctc.9b00969. 31670947

[ref34] Iino T.; Shiozaki T.; Yanai T. Algorithm for analytic nuclear energy gradients of state averaged DMRG-CASSCF theory with newly derived coupled-perturbed equations. J. Chem. Phys. 2023, 158 (5), 054107 10.1063/5.0130636. 36754810

[ref35] Kim S. Y.; Park J. W. Approximate Excited-State Geometry Optimization with the State-Averaged Adaptive Sampling Configuration Interaction Algorithm with Large Active Spaces. J. Chem. Theory Comput. 2023, 19, 7260–7272. 10.1021/acs.jctc.3c00808. 37800852

[ref36] Ugandi M.; Roemelt M. A configuration-based heatbath-CI for spin-adapted multireference electronic structure calculations with large active spaces. J. Comput. Chem. 2023, 44, 2374–2390. 10.1002/jcc.27203. 37589287

[ref37] Ugandi M.; Roemelt M. A recursive formulation of one-electron coupling coefficients for spin-adapted configuration interaction calculations featuring many unpaired electrons. Int. J. Quantum Chem. 2023, 123, e27045 10.1002/qua.27045.

[ref38] Helgaker T.; Jorgensen P.; Olsen J. Molecular electronic-structure theory; John Wiley & Sons, 2013.

[ref39] Helmich-Paris B. A trust-region augmented Hessian implementation for state-specific and state-averaged CASSCF wave functions. J. Chem. Phys. 2022, 156 (20), 204104 10.1063/5.0090447. 35649854

[ref40] Chilkuri V. G.; Neese F. Comparison of many-particle representations for selected-CI I: A tree based approach. J. Comput. Chem. 2021, 42, 982–1005. 10.1002/jcc.26518. 33764585

[ref41] Chilkuri V. G.; Neese F. Comparison of many-particle representations for selected configuration interaction: II. Numerical benchmark calculations. J. Chem. Theory Comput. 2021, 17, 2868–2885. 10.1021/acs.jctc.1c00081. 33886300 PMC8279407

[ref42] Huron B.; Malrieu J.; Rancurel P. Iterative perturbation calculations of ground and excited state energies from multiconfigurational zeroth-order wavefunctions. J. Chem. Phys. 1973, 58, 5745–5759. 10.1063/1.1679199.

[ref43] Tubman N. M.; Freeman C. D.; Levine D. S.; Hait D.; Head-Gordon M.; Whaley K. B. Modern approaches to exact diagonalization and selected configuration interaction with the adaptive sampling CI method. J. Chem. Theory Comput. 2020, 16, 2139–2159. 10.1021/acs.jctc.8b00536. 32159951

[ref44] Smith J. E.; Lee J.; Sharma S. Near-exact nuclear gradients of complete active space self-consistent field wave functions. J. Chem. Phys. 2022, 157 (9), 094104 10.1063/5.0085515. 36075733

[ref45] Holmes A. A.; Umrigar C. J.; Sharma S. Excited states using semistochastic heat-bath configuration interaction. J. Chem. Phys. 2017, 147 (16), 164111 10.1063/1.4998614. 29096501

[ref46] Helgaker T.; Jørgensen P. Configuration-interaction energy derivatives in a fully variational formulation. Theor. Chim. Acta 1989, 75, 111–127. 10.1007/BF00527713.

[ref47] Bernhardsson A.; Lindh R.; Olsen J.; Fulscher M. A direct implementation of the second-order derivatives of multiconfigurational SCF energies and an analysis of the preconditioning in the associated response equation. Mol. Phys. 1999, 96, 617–628. 10.1080/00268979909482998.

[ref48] Davidson E. R. The iterative calculation ofafew ofthe lowest eigenvalues and corresponding eigenvectors oflarge real-symmetric matrices. J. Comput. Phys. 1975, 17, 87–94. 10.1016/0021-9991(75)90065-0.

[ref49] Liu B.; The simultaneous expansion method for the iterative solution of several of the lowest-lying eigenvalues and corresponding eigenvectors of large real-symmetric matrices. In Numerical Algorithms in Chemistry: Algebraic Methods; Lawrence Berkeley Laboratory, 1978. 49–53.

[ref50] Valeyev E., evaleev/libint: 2.8.0; Zenodo, 2023.

[ref51] Siegbahn P. E.; Almlöf J.; Heiberg A.; Roos B. O. The complete active space SCF (CASSCF) method in a Newton–Raphson formulation with application to the HNO molecule. J. Chem. Phys. 1981, 74, 2384–2396. 10.1063/1.441359.

[ref52] Sun Q.; Yang J.; Chan G. K.-L. A general second order complete active space self-consistent-field solver for large-scale systems. Chem. Phys. Lett. 2017, 683, 291–299. 10.1016/j.cplett.2017.03.004.

[ref53] Kreplin D. A.; Knowles P. J.; Werner H.-J. Second-order MCSCF optimization revisited. I. Improved algorithms for fast and robust second-order CASSCF convergence. J. Chem. Phys. 2019, 150 (19), 194106 10.1063/1.5094644. 31117783

[ref54] Kreplin D. A.; Knowles P. J.; Werner H.-J. MCSCF optimization revisited. II. Combined first-and second-order orbital optimization for large molecules. J. Chem. Phys. 2020, 152 (7), 074102 10.1063/1.5142241. 32087666

[ref55] Damour Y.; Véril M.; Kossoski F.; Caffarel M.; Jacquemin D.; Scemama A.; Loos P.-F. Accurate full configuration interaction correlation energy estimates for five-and six-membered rings. J. Chem. Phys. 2021, 155 (13), 134104 10.1063/5.0065314. 34624964

[ref56] Yao Y.; Umrigar C. Orbital optimization in selected configuration interaction methods. J. Chem. Theory Comput. 2021, 17, 4183–4194. 10.1021/acs.jctc.1c00385. 34132530

[ref57] Høyvik I.-M.; Jansik B.; Jørgensen P. Trust region minimization of orbital localization functions. J. Chem. Theory Comput. 2012, 8, 3137–3146. 10.1021/ct300473g. 26605725

[ref58] Helmich-Paris B. A trust-region augmented Hessian implementation for restricted and unrestricted Hartree–Fock and Kohn–Sham methods. J. Chem. Phys. 2021, 154 (16), 164104 10.1063/5.0040798. 33940809

[ref59] Fletcher R. Practical methods of optimization; John Wiley & Sons, 2000.

[ref60] Feldmann R.; Baiardi A.; Reiher M. Second-order self-consistent field algorithms: from classical to quantum nuclei. J. Chem. Theory Comput. 2023, 19, 856–873. 10.1021/acs.jctc.2c01035. 36701300

[ref61] Park J. W. Second-order orbital optimization with large active spaces using adaptive sampling configuration interaction (ASCI) and its application to molecular geometry optimization. J. Chem. Theory Comput. 2021, 17, 1522–1534. 10.1021/acs.jctc.0c01292. 33630610

[ref62] Baerends E. J.; Ellis D.; Ros P. Self-consistent molecular Hartree-Fock-Slater calculations I. The computational procedure. Chem. Phys. 1973, 2, 41–51. 10.1016/0301-0104(73)80059-X.

[ref63] Dunlap B. I.; Connolly J. W.; Sabin J. R. On the applicability of LCAO-X*α* methods to molecules containing transition metal atoms: The nickel atom and nickel hydride. Int. J. Quantum Chem. 1977, 12, 81–87. 10.1002/qua.560120813.

[ref64] Dunlap B.; Connolly J.; Sabin J. On first-row diatomic molecules and local density models. J. Chem. Phys. 1979, 71, 4993–4999. 10.1063/1.438313.

[ref65] Kendall R. A.; Früchtl H. A. The impact of the resolution of the identity approximate integral method on modern ab initio algorithm development. Theor. Chem. Acc. 1997, 97, 158–163. 10.1007/s002140050249.

[ref66] Werner H.-J.; Manby F. R.; Knowles P. J. Fast linear scaling second-order Møller-Plesset perturbation theory (MP2) using local and density fitting approximations. J. Chem. Phys. 2003, 118, 8149–8160. 10.1063/1.1564816.

[ref67] Hohenstein E. G.; Sherrill C. D. Density fitting and Cholesky decomposition approximations in symmetry-adapted perturbation theory: Implementation and application to probe the nature of π-π interactions in linear acenes. J. Chem. Phys. 2010, 132 (18), 184111 10.1063/1.3426316.

[ref68] Beebe N. H.; Linderberg J. Simplifications in the generation and transformation of two-electron integrals in molecular calculations. Int. J. Quantum Chem. 1977, 12, 683–705. 10.1002/qua.560120408.

[ref69] Aquilante F.; Boman L.; Boström J.; Koch H.; Lindh R.; de Merás A. S.; Pedersen T. B. Cholesky decomposition techniques in electronic structure theory Linear-Scaling Techniques In Computational Chemistry And Physics: Methods And Applications Springer 2011 13 301–343 10.1007/978-90-481-2853-2_13

[ref70] Pedersen T. B.; Lehtola S.; Fdez. galván I.; Lindh R. The versatility of the Cholesky decomposition in electronic structure theory. Wiley Interdiscip. Rev.: comput. Mol. Sci. 2024, 14, e1692 10.1002/wcms.1692.

[ref71] Kossmann S.; Neese F. Efficient structure optimization with second-order many-body perturbation theory: The RIJCOSX-MP2 method. J. Chem. Theory Comput. 2010, 6, 2325–2338. 10.1021/ct100199k. 26613489

[ref72] Izsák R.; Neese F. An overlap fitted chain of spheres exchange method. J. Chem. Phys. 2011, 135 (14), 144105 10.1063/1.3646921. 22010696

[ref73] Song C.; Martínez T. J.; Neaton J. B. A diagrammatic approach for automatically deriving analytical gradients of tensor hyper-contracted electronic structure methods. J. Chem. Phys. 2021, 155 (2), 024108 10.1063/5.0055914. 34266268

[ref74] Bangerter F. H.; Glasbrenner M.; Ochsenfeld C. Tensor-Hypercontracted MP2 First Derivatives: Runtime and Memory Efficient Computation of Hyperfine Coupling Constants. J. Chem. Theory Comput. 2022, 18, 5233–5245. 10.1021/acs.jctc.2c00118. 35943450 PMC9476664

[ref75] Wex B.; Kaafarani B. R. Perspective on carbazole-based organic compounds as emitters and hosts in TADF applications. J. Mater. Chem. C 2017, 5, 8622–8653. 10.1039/C7TC02156A.

[ref76] Servan S. A.; Unal A.; Hamarat B.; Bozkaya U. Assessment of the Density-Fitted Second-Order Quasidegenerate Perturbation Theory for Transition Energies: Accurate Computations of Singlet–Triplet Gaps for Charge-Transfer Compounds. J. Phys. Chem. A 2020, 124, 6889–6898. 10.1021/acs.jpca.0c04555. 32786988

[ref77] Noirbent G.; Xu Y.; Bonardi A.-H.; Duval S.; Gigmes D.; Lalevée J.; Dumur F. New donor-acceptor Stenhouse adducts as visible and near infrared light polymerization photoinitiators. Molecules 2020, 25, 2317 10.3390/molecules25102317. 32429126 PMC7287840

[ref78] Khedkar A.; Roemelt M. Active space selection based on natural orbital occupation numbers from n-electron valence perturbation theory. J. Chem. Theory Comput. 2019, 15, 3522–3536. 10.1021/acs.jctc.8b01293. 31059643

[ref79] Khedkar A.; Roemelt M. Extending the ASS1ST active space selection scheme to large molecules and excited states. J. Chem. Theory Comput. 2020, 16, 4993–5005. 10.1021/acs.jctc.0c00332. 32644789

[ref80] Pracht P.; Bohle F.; Grimme S. Automated exploration of the low-energy chemical space with fast quantum chemical methods. Phys. Chem. Chem. Phys. 2020, 22, 7169–7192. 10.1039/C9CP06869D. 32073075

[ref81] Grimme S. Exploration of chemical compound, conformer, and reaction space with meta-dynamics simulations based on tight-binding quantum chemical calculations. J. Chem. Theory Comput. 2019, 15, 2847–2862. 10.1021/acs.jctc.9b00143. 30943025

[ref82] Angeli C.; Cimiraglia R.; Malrieu J.-P. n-electron valence state perturbation theory: A spinless formulation and an efficient implementation of the strongly contracted and of the partially contracted variants. J. Chem. Phys. 2002, 117, 9138–9153. 10.1063/1.1515317.

[ref83] Roemelt M.; Krewald V.; Pantazis D. A. Exchange Coupling Interactions from the Density Matrix Renormalization Group and N-Electron Valence Perturbation Theory: Application to a Biomimetic Mixed-Valence Manganese Complex. J. Chem. Theory Comput. 2018, 14, 166–179. 10.1021/acs.jctc.7b01035. 29211960

[ref84] Neese F. The ORCA program system. Wiley Interdiscip. Rev.: comput. Mol. Sci. 2012, 2, 73–78. 10.1002/wcms.81.

[ref85] Neese F.; Wennmohs F.; Becker U.; Riplinger C. The ORCA quantum chemistry program package. J. Chem. Phys. 2020, 152 (22), 224108 10.1063/5.0004608. 32534543

[ref86] Dunning Jr T. H. Gaussian basis sets for use in correlated molecular calculations. I. The atoms boron through neon and hydrogen. J. Chem. Phys. 1989, 90, 1007–1023. 10.1063/1.456153.

[ref87] Weigend F.; Ahlrichs R. Balanced basis sets of split valence, triple zeta valence and quadruple zeta valence quality for H to Rn: Design and assessment of accuracy. Phys. Chem. Chem. Phys. 2005, 7, 3297–3305. 10.1039/b508541a. 16240044

[ref88] Weigend F. Hartree–Fock exchange fitting basis sets for H to Rn. J. Comput. Chem. 2008, 29, 167–175. 10.1002/jcc.20702. 17568435

[ref89] Fdez. Galvan I.; Delcey M. G.; Pedersen T. B.; Aquilante F.; Lindh R. Analytical state-average complete-active-space self-consistent field nonadiabatic coupling vectors: Implementation with density-fitted two-electron integrals and application to conical intersections. J. Chem. Theory Comput. 2016, 12, 3636–3653. 10.1021/acs.jctc.6b00384. 27327873

[ref90] Coe J. P. Analytic Non-adiabatic Couplings for Selected Configuration Interaction via Approximate Degenerate Coupled Perturbed Hartree–Fock. J. Chem. Theory Comput. 2023, 19, 8053–8065. 10.1021/acs.jctc.3c00601. 37939698 PMC10687870

[ref91] Siegbahn P.; Heiberg A.; Roos B.; Levy B. A comparison of the super-CI and the Newton-Raphson scheme in the complete active space SCF method. Phys. Scr. 1980, 21, 323 10.1088/0031-8949/21/3-4/014.

