# Supplementary material for: Analytical SA-HCISCF Nuclear Gradients from Spin-Adapted Heat-Bath Configuration Interaction
Source: J Chem Theory Comput. 2025 Apr 7;21(8):3930–44. doi: 10.1021/acs.jctc.5c00021 (PMC12020362; doi:10.1021/acs.jctc.5c00021)
Supplement: Supplementary file 1 — ct5c00021_si_001.pdf [file ct5c00021_si_001.pdf]

Supporting Information for "Analytical SA-HCISCF Nuclear Gradients from Spin-Adapted Heat-bath Configuration Interaction"

Mihkel Ugandi and Michael Roemelt*

Institut für Chemie, Humboldt-Universität zu Berlin, Brook-Taylor-Str. 2, D-12489 Berlin, Germany

E-mail: michael.roemelt@hu-berlin.de

ASS1ST orbital selection

A1 singlet

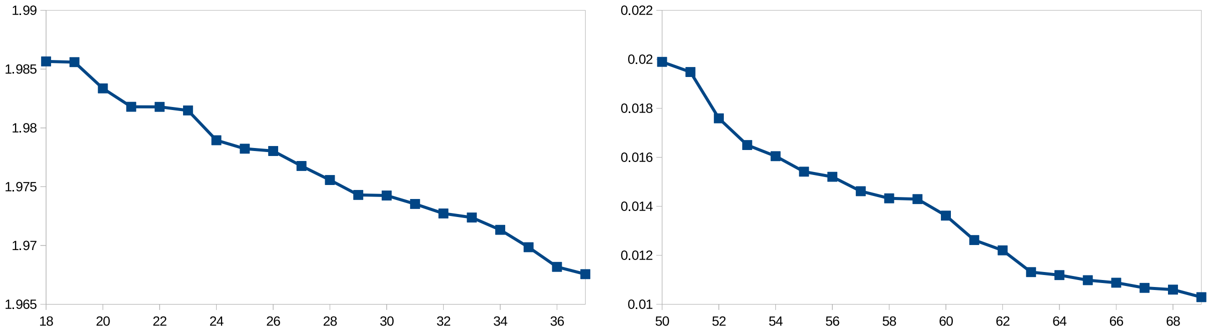

Figure S1: External and internal ASS1ST(12e, 12o)/cc-pVDZ pseudonatural orbitals for A1 (singlet).

A1 doublet

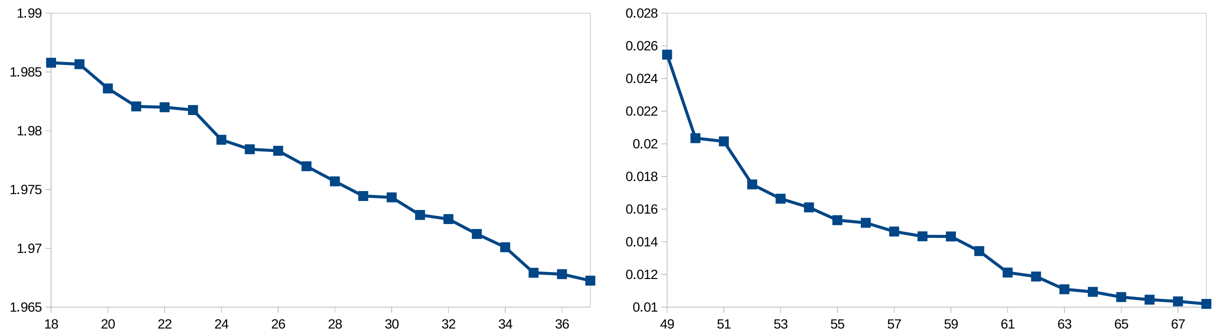

Figure S2: External and internal ASS1ST(11e, 11o)/cc-pVDZ pseudonatural orbitals for A1 (doublet).

A1 triplet

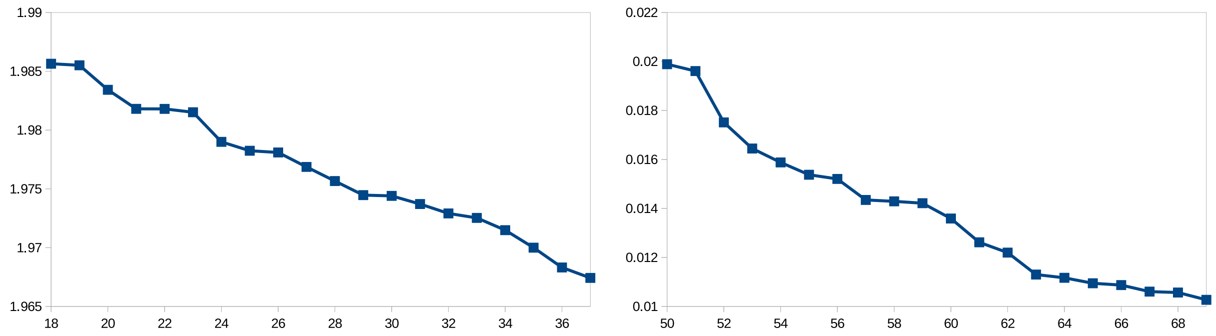

Figure S3: External and internal ASS1ST(12e, 12o)/cc-pVDZ pseudonatural orbitals for A1 (triplet).

A2 singlet

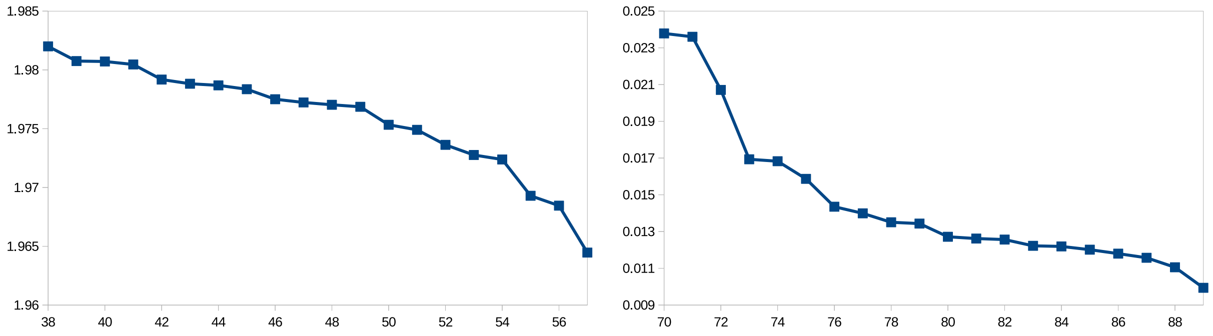

Figure S4: External and internal ASS1ST(12e, 12o)/def2-SVP pseudonatural orbitals for A2 (singlet).

A2 triplet

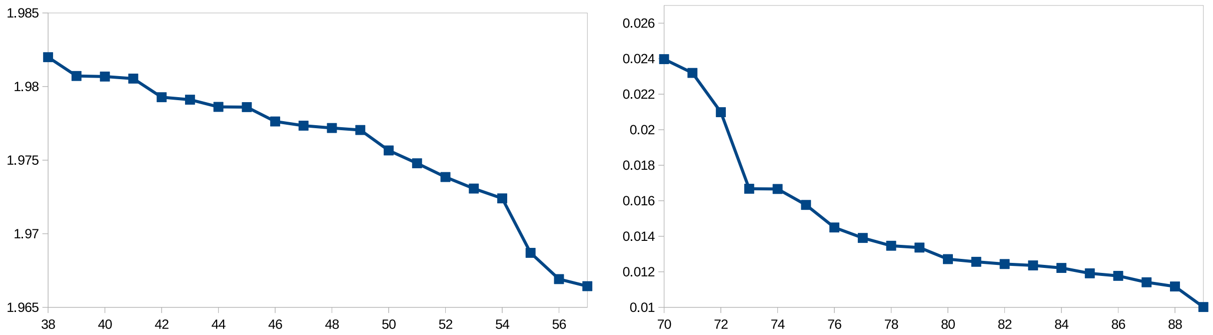

Figure S5: External and internal ASS1ST(12e, 12o)/def2-SVP pseudonatural orbitals for A2 (triplet).

A3 singlet

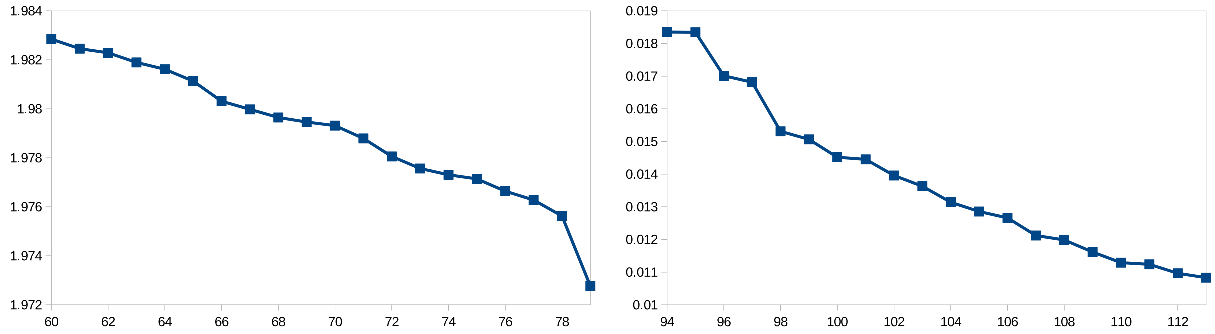

Figure S6: External and internal ASS1ST(12e, 12o)/def2-SVP pseudonatural orbitals for A3 (singlet).

B1 triplet

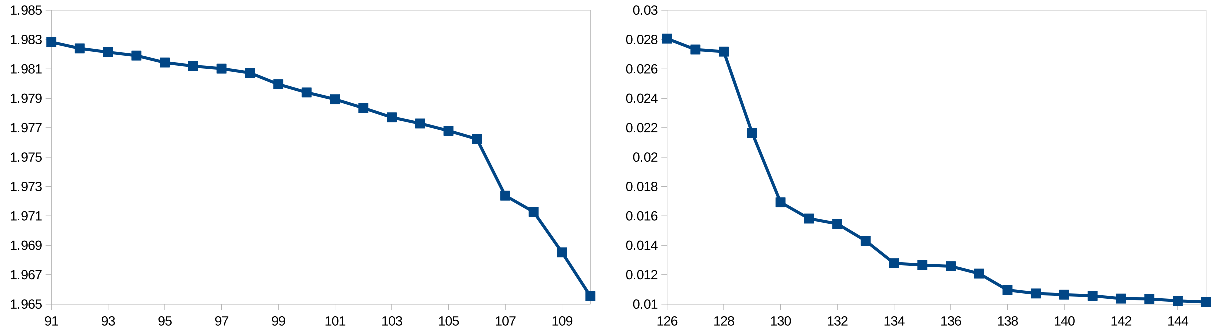

Figure S7: External and internal ASS1ST(16e, 15o)/def2-SVP pseudonatural orbitals for B1 (triplet).
